# Slouching toward New Humanism

**DOI:** 10.3389/fsoc.2023.1111690

**Published:** 2023-10-19

**Authors:** Paolo Contini, Elisabeta Osmanaj

**Affiliations:** ^1^University of Bari Aldo Moro, Bari, Italy; ^2^Centro Interuniversitario di Ricerca “Popolazione, Ambiente e Salute”, Bari, Italy; ^3^Universiteti Aleksander Xhuvani, Elbasan, Albania

**Keywords:** Humanism, postmodernity (postmodern condition), augmented anthropology, New Humanism, sociology, ethics

## Abstract

In the contemporary era, novel social and cultural patterns have emerged, prompting the social sciences to engage in timely reflections on current phenomena and the very essence of humanity. These models have undergone significant transformations, so much so that New Humanism appears to be a viable prospect. It is upon this premise that all the observations put forth in this study are centered, operating under the assumption that the evolution of knowledge is a continuous process, marked by perpetual paths of research and reflection. It is well established that Humanism represents a recurring theme in our daily lives, with its premises inscribed on the walls of cities and subways. In the modern era, the concept of Humanism is liberated from its “classical” meanings. As such, it becomes crucial to consider New Humanism as a supplement for the soul, capable of invigorating spirituality, restoring energy, and instilling ethics rooted in solidarity, recognition, and mutual respect among individuals. This mission serves as a formidable catalyst, motivating and mobilizing the consciences and intelligence of individuals, particularly those in the Western world. Furthermore, this pressing need necessitates the adoption of conceptualization and analytical reconstruction pathways, which are functional in actualizing the perspective of New Humanism, establishing it as a proposition of contemporary culture. It is often implicit in widespread individualism, which tends to manifest exaggerated and exasperating tones of hyper-individualism. It is thus essential not to overlook the doctrine espoused by classical Humanism, which suggests that it is possible to be disenchanted and yet remain builders of utopias, and realists who can see new lands and infinite ideal worlds. Indeed, human beings can transcend existing barriers, using tradition as a viaticum for the future, as evidenced by numerous disciplinary fields.

## 1. Introduction

The advent of the 21^st^ century marks a new season full of unprecedented social and cultural patterns, in the context of which social sciences are called to provide an appropriate reflection of both the current phenomena and the man himself (Cesareo, 2019a). These patterns have been strongly affected by change, so much to allow us to speak of the birth of New Humanism. Hence, all the observations proposed here focus on New Humanism, proceeding from the assumption that the evolution of knowledge passes through the never-ending paths of research and reflection.

It is well known that Humanism represents a recurring phenomenon, conveyed to us as a fact present in our daily life—the premises of which are written on the walls of cities and subways—and devoid of its “classical” meanings as a result of modernity. Once the convictions about the ultimate foundations of reality get destroyed, it becomes crucial to think of New Humanism as equipped with a soul supplement, namely, means capable of reviving the latter spiritually, restoring its energy, and embedding it deeply on the ethical ground of solidarity, recognition, and mutual respect among fellow human beings. Such a mission represents a formidable factor that motivates and mobilizes consciences and intelligences, particularly those of the West (Cesareo, 2019b).

Moreover, this need urges the undertaking of paths of conceptualization and analytical reconstruction functional to actualize the perspective of New Humanism, defining it as a proposal of contemporary culture, often implied by a widespread individualism that tends to take on the exaggerated and exasperating tones of hyper-individualism.^1^

At the same time, these paths cannot ignore and consign to oblivion the past from which they derive; this would risk what Guicciardini has well-explained in his book *Ricordi politici e civili*, where he points out that “the past gives light to the future, for the world has always been of the same lot; and all that is, and will be, existed in another time, and the same things repeat themselves but under changed names and colors; yet no one recognizes them, with the exception of those who are wise and observe them and consider them diligently” (Guicciardini, 1933, p. 263). The words of a scholar like Guicciardini echo those of the mathematician Poincaré who emphasized the same concept by arguing that “we do not study the starry sky and its laws in order to find new laws with which we can build new machines, but we are always creating new machines so that an increasing number of men, relieved from physical labor, can freely investigate the sky” (Poincaré, 1992).

We cannot leave without mentioning the corpus of lessons that we have learned from classical Humanism, according to which it is possible to be disenchanted and become, at the same time, builders of utopias and realists capable of seeing new lands, new heavens (Galilei), as well as of reading the infinite ideal worlds (Giordano Bruno). The human being can transcend existing barriers by celebrating tradition as a viaticum for the future, as is the case in many disciplinary fields (Ciliberto, 2017).

It is with Renaissance Humanism that man is placed at the center of the world in a markedly anthropocentric vision, iconically represented by Leonardo's Vitruvian Man. By focusing attention on the great possibilities of man and concentrating on the almost absolute value of education as the privileged path for the development of every possibility, the foundations are laid for the great transformations that will lead to the Enlightenment and the Reformation.

The renewed actuality of Humanism is related to techno-capitalism and its innovative responses to the complex and unresolved questions inherent in humanity. From this point of view, man is reconsidered from the perspective of augmented anthropology, which proposes the elaboration of a technological Humanism characterized by numerous unprecedented challenging questions, whose possible (and provisional) answers bring to the fore new priorities that, in the recent past, have been considered of little importance. Moreover, the same thinking considers the logic permeating new technical and technological devices as a danger, raising the question of what might be an available guiding criterion. Technological Humanism, together with the foreshadowing of its planetary extensions and lasting consequences on humanity, raises questions related to the ethical principles regarding the new perspectives of *enhanced man*.

If the *heuristics of fear* is avoided, the upheaval of man may encourage the reconceptualization of humanity, preserving it from the danger of the artifactualization of the world (Benasayag, 2016a). Being aware of the fact that what is at stake—as anticipated a few years ago by Jonas (2002)—is not only human destiny but the very image of man; not only the physical survival but the integrity of the being and of his ethics itself, which has the function of safeguarding both dimensions by combining prudence with respect (the Kantian *Ehrfurcht*).

Ultimately, the problem of the human condition, an open question that has become tragic due to the entirely new forms proposed by *transhumanism* and *post-humanism*, has arisen again. We are being faced with a difficult task concerning the current societal condition, which is characterized by complex socio-cultural phenomena that, as we have anticipated, find expression in historicization and contextualization in modernity; but all that does not imply exception from the assumed reflection on Humanism and its new forms *in progress*.^2^

Unlike previous Humanism, which was characterized by its ideal of a man^3^, by the idea of achievement focused on human greatness, capable of striving with reason and will toward “Infinite Mystery” (masterfully personified by Dante's Ulysses, Canto XXVI of *Inferno*), current Humanism has the traits of a self-referential and fragmented identity, and at the same time individualistic and narcissistic, lacking historical consciousness, and therefore absorbed in the present moment. These are the distinguishing features of exclusive Humanism that, along with its obtuse particularity on which it is based, are generally insufficient for themselves.

The same drift in identity, which in the past used to manifest itself as a phenomenon confined to individuals, is now transforming into a phenomenon with significant implications for collective life as a result of the growing expansion of individualism and its emergence of attitudes, orientations, and behaviors that are mostly unprecedented in the history of Western societies.

This explains why the anthropological question (or rather, anthropocentric excess), which regards reality as the ground where man exercises his sovereignty, constitutes the real social issue of contemporary times. Anthropocentrism has been one of the central goals of modernity aimed at giving more space to the consideration of man as the subject of the world and as the builder of his destiny (homo faber suae fortunae). However, this operation has proved to be detrimental to the human being itself due to the destruction wrought against its truest dimension, the most concrete essence of its being, fullness, and finality in itself. According to Magatti, such an anthropocentric drift has caused, and continues to cause, distortions not only to humanity but also to the relationship between human beings and their ecosystem (Magatti and Giaccardi, 2019).^4^

Hence, the call for a change of pace, for a rediscovery of the history of mankind and its deepest identity, has recently been articulated in a codified proposal of anthropomorphic conception (Marchesini, 2016). This aims to reclaim the wholeness of human life and to give a fulfilled meaning to the unconscious life, which is considered part of a common world and a whole being, belonging to the same teleological structure of human existence. Such a structure can only be grasped and appropriately respected by humans by virtue of reason.

## 2. New Humanism: what anthropology of postmodernity?

For all the aforementioned reasons, we cannot conceive of New Humanism outside of a semantic framework and qualitative distinctions regarding the conception of man and the world (e.g., individual-person), transitioning from a philosophical to an instrumental dimension (Magatti, 2018a).

To gain an understanding of the current situation, it is necessary to revisit modernity, a phase we have moved past, characterized by a Humanism that has achieved significant progress in terms of welfare, democracy, freedom, awareness, and, last but not least, technological/technical advancement, capable of bringing about unprecedented changes.

The advanced technological levels have had an impact on institutional and organizational assets on one hand and cultural and cognitive aspects on the other. While systems and infrastructures have become global, efficient, and high-performing, at the cultural level, change has affected the human being, perceived as an abstract, fragmented individual, separated not only from the reality that surrounds them but even from their loved ones. As a result, digital technologies, along with their grammar and their pursuit of flexibilization, virtualization, and rationalization goals (e.g., of work), are shaping worldviews and human conceptions (Beck, 2000).

Furthermore, they convey the illusion of the rediscovery of man, the homo tecnologicus, considered to be the distinguishing figure of the post-modern, post-financial, and consumer society. This type of man is assumed to be the measure of all things, a viewpoint that had already been denounced by Nietzsche as erroneous insofar as it is based on the assumption that things are immediately given to man as pure objects. Such a viewpoint supports a conception whose core assumption is that everything is built to the measure of man, and hence, a world where there is too much of man: a choice justified by the quest for strength/power, efficiency, and impersonality, but where in reality, there is no longer any place for the human being.

Thus, New Humanism cannot ignore this all-encompassing vision, nor the lessons from the recent past. It must engage in the deconstruction of the conceptual system conveyed by the current perspective and seek an appreciation of the rich and profound traits of the human being without yielding to the emphasis on the construction of an ultra-human being, i.e., the enhanced man. To proceed in this way, there must be a clarification of the anthropological option and a substantive vision of man, rather than just a simple posture oriented toward improvement or physical survival (Jonas, 2002).^5^ It is essential to be aware of the fact that the fate of humanity is at stake.

It is important to defend and propose an ideal focused on valuing the integrity of being and the person themselves, as pointed out in the opening of the paragraph.^6^ This option is supported by numerous reasons, which we will explore in this study, including historical and theoretical reasons (Cesareo, 2019a).

The historical reason for valuing the integrity of being and the person is rooted in the course of human mutations. A particular type of human being is emerging: for some, the minimalist narcissist (Cesareo, 2019c), and for others, the individualist. They are united, however, by a set of defining characteristics that are the exact opposite of what it means to be human.

The individual has represented the ideal of a man conforming to the modern social order, with characteristics suited to the needs of industrial production and the developed capitalist economy. Habermas, one of the scholars who has studied modern individualism, believes that it is a topos related to an ideal of man negatively affected by dominant stereotypes, oriented toward a repertoire of socially conveyed models of action, and aimed at pursuing an identity focused on self-interest (Habermas, 2003).

In contrast, Norbert Elias focuses on the social mechanisms that have promoted varying degrees of individualization in modernity, and how the individual gains autonomy from the social group and succeeds in expressing their originality. Regarding this, he writes:

It is only after being socially shaped within the framework of certain social-typical personality traits that personality traits and modes of behavior, by which human beings differ from one another within a society, are formed. *Society not only has the function of equalizing and typifying, but also that of individualizing*, as shown by the different degree of individualization of the members of various groups and strata (Elias, 1990, p. 75).

The question then arises whether, in the presence of individualism, which is the exact opposite of the individual but a socially pursued ideal, it is still possible to bring the person back to the center of the social scene. This question leads us to theoretical reasoning and hence to a reflection on the individual and their distinctive traits.

We consider it appropriate to start with Nietzsche and his conception of humanistic civilization centered on man, which he considers radically unsuccessful as it focuses on the promise of perfection, while the human being is still far from it. He blames humanistic civilization, supported by ancient Greek culture (Platonic) and Christian civilization (modern), for distracting humanity from the very human goal of governing the world and guiding man toward a spiritual object (e.g., Platonic Hyperuranium or the Christian Kingdom of Heaven). According to Nietzsche, we must remain faithful to the earth and not believe those who speak of extraterrestrial hopes and human transcendentality.

The concept of man as a creature of God was the ideal of classical humanism, a masterfully formalized conception, as we have anticipated, in the thought of Dante (medieval culture) and in the song of Ulysses, where the theme of human potential realization is clarified.^7^ The Homeric hero represents humanity facing the unknown with only human forces and is the exaltation of the greatness of man capable of tending toward the “infinite Mystery”^8^ with the help of reason and will.

Renaissance humanism, on the other hand, the forerunner of modernity, proposes a different way of understanding human potential realization: man becomes fulfilled through his own forces, independently of God, who, even though not denied, is no longer considered necessary for the success of man.

Currently, as in the past, the greatest discovery remains man, seen as a social actor and a real force on which to build the post-modern, post-financialized economy. Current Humanism is driven by the fluctuation of previous categories, which are considered no longer suitable to decode and cope with the changes taking place, and to provide suitable tools to rethink the ideal of man. This approach places great confidence in man and his openness/possibility to change or, as is already being advocated, to *enhancement*.

In view of the possible achievement of the realization of this model, an in-depth reflection on the human being and his prospects is urgently needed.^9^ As mentioned previously, the current phase is characterized by great changes, made difficult by epochal transformations related to immigration, wars, advanced technologies and their applications in the field of science, and more. These transformations involve cultural roots and identities and require new tools to deal with change and its consequences, such as rethinking the human ideal and developing critical categories suitable for interpreting questions and seeking answers.

In this scenario, the role of New Humanism is fundamental, as it can serve as an instrument for collective cultural growth. To this end, while not ignoring its ties with tradition, it can provide depth to the scientific-technological field due to its pedagogical value.^10^

Moreover, among the dilemmatic issues which New Humanism has to face, there are the real social problems of the new century: the protection of human integrity and sustainability (environmental, economic, etc.). These areas involve the value sphere, which has progressively been differentiated and autonomized, similar to what happened to awareness, and requires a new reworking space as well. New Humanism, following an all-encompassing vision of the whole reality and of the human being understood as an individual endowed with a wealth of meaning, not yet characterized by a miserable and banal condition (Taylor, 1993), constitutes an area where integrity and sustainability can be preserved both at a personal level (life, family, and education) and social level, protecting the backbone of social life (justice, solidarity, and work).

The person is referred to as a unique, historical, concrete, and relational being, different from the individual who identifies man through his anatomical and physiological components. Vincenzo Cesareo, the Italian sociologist who has delved deeper into this field, points out that the distinctive character of the person is his distancing from the conceptual models of man centered on the opposition of the primacy of corporeity or rationality and develops a perspective that takes as reference the distinctive characteristics of uniqueness and human transcendentality. The scholar recognizes the person's ability to reorient his actions in a functional way toward life, not bending them to his own interests, and asks questions about man, his ontological consistency, and what makes men equal and different at the same time (Cesareo and Vaccarini, 2006).

At the international level, Robert Spaemann is the most representative sociologist of the studies on the individual. According to him, the human being represents an authentic idea of Western culture: “People, among all that exists, occupy a particular position. Taken together, they do not constitute a natural species. In order to know whether we are dealing with ‘something' or ‘someone,' we need to know what kind of being it is” (Spaemann, 2016). The philosopher also introduces the delicate issue of the position occupied by the individual as a result of his uniqueness and not as a result of any other form of existence taken up by the current debate. Unlike other living species, the individual exists, and it is this characteristic that distinguishes the being from its existential determinations (the ways of being) and from its determined nature. Even if the individual acknowledges the negative implications of the distance that he can establish with himself, Spaemann reiterates that this does not diminish his uniqueness, but rather further testifies to it.

With this, he points out that the person is not comparable to everything that exists and requires a specific reflection because his definition does not end up in the objective description of what he is: he is not a thing, but a who. This who is not reducible to the idea of subjectivity. Every individual not only sees and perceives himself but is also seen and perceived by others; hence, he is also exteriority, which presupposes “a plurality of people for whom there is an externality of this individual” (Sabangu, 2005).

Recently, the concept of the individual has been questioned again by the empiricist and neuroscience approaches, united by a reductionist vision of man. Neurosciences cause man and his actions to collapse over the activity of the central nervous system, bypassing man's prerogative to exist (Spaemann, 2016), being able to transcend oneself because of the possession of specific and distinctive characteristics among all living beings, such as responsibility (toward the natural environment, etc.), the ability to distance oneself from oneself, and to reflect. Even the empiricist approach adopts a reductionism, leading the complex reality of the individual to a single property, to a character that constitutes only one aspect: the psychic states, an assumption that contains something true, but not the whole truth, as it does not grasp the distinctive specificity of the human species compared to the others. Empiricists concentrate on the identification of a general quality shared by existing beings, by every animal that has a certain degree of self-consciousness and mnemonic ability, assuming it as a determinative factor of the character of the individual.

What is outlined represents the horizon where New Humanism is inscribed and the background to the longstanding questions posed by recent cultural developments, on which we will dwell in the following pages.

## 3. Technological humanism: *Transhumanism* and *post-humanism*

One of the reasons (historical and theoretical) that animates the contemporary debate is the anthropological mutation of man, which has transitioned from individualism to minimalist narcissism, a form of self-referential privatization of things and the alternate self (Cesareo, 2019c).

Individualism has reformulated the idea of man, making viable the particularistic and one-dimensional visions of the human being (e.g., narcissism) and removing the complex horizons of the sense of modern Humanism. In this way, it has conveyed a radically simplified and reductive vision of man and reality (Fornari, 2014, p. 6). This anthropological conception is focused on an auto-centric identity and a bio-psychological organism characterized by impulses and needs that demand immediate satisfaction with the least effort. It is a representation that is located outside the horizon of the person, who is denied any possibility of being at the center of today's social scene.^11^

The result is the affirmation of an ontology centered on the ideal of a pure subject, in which the body no longer refers to subjectivity characterized by uniqueness generated by the union of several aspects, but rather as a simple pre-ordered biomechanical structure, an object of technological manipulations (Fornari, 2014). Based on this, the body is taken for granted, and the complete identification between man and technology is pursued. This identification is widely followed due to the provision of large and efficient infrastructures by technology, and it consolidates man's desire for power, which is now exercised through the manipulation of reality.

Although technology indeed brings progress and planetary connection, it also leads to the decomposition and fragmentation of reality, making use of man's sense of power. Bauman (2018) argues that men are launched into this new adventure of removing any limit, any constraint, and any organic regulation, believing that without regulations and limits, total freedom belongs to us and cannot be just a mere promise.^12^

In line with the thinking of the Polish sociologist, Benasayag argues that there is a “temptation of unlimited power” increasingly accompanied by the “promise of total deregulation,” which stands in clear opposition to “the very essence of life in all its dimensions: fragility.” Fragility should not be understood as a weakness but as the transience of life, memory, and the realization of nothingness. The current culture is the first to be possessed by technology, which has generated the idea of living in an era where everything is possible and where what seems impossible is, in reality, interpreted as not yet possible (Benasayag, 2016b, 2019b).

This idea of power makes the problematic issues introduced by this techno-economic ideology secondary, focusing on an abstract, icy, and detached dogmatism: breakdown and fragmentation, and therefore, a corporate ideal made of isolated atoms, possibly neutral, autonomous, and functioning, organized by extended and performing systems, which meet occasionally and temporarily for an exchange of interest or mutual enjoyment (e.g., Exclusive humanism; Magatti, 2018b).

The technical culture, which is being structured within an increasingly technological universe, permeates mental representations, and models of action, prefiguring an ideal of homo technologicus. The latter is understood as a hybrid evolutionary unit, a symbiont in continuous transformation, a product of technological fascination, and the spread of extended and performing systems. Such systems undergo a transition from occasional and temporary presences to pervasive factors of individual and collective biographies, from operational tools to extensions of human abilities aimed at increasing physical and intellectual abilities, if not control over life.^13^

Therefore, technology constitutes the environment in which *Homo technologicus* is immersed. It dissolves the previous conception of man and spreads the illusion—or, if you will, the hope—of the domination of technology over existence. The consequence is the progressive disappearance of human subjectivity, replaced by an individuality understood as a computational-representational system, a depiction to which neuroscience has contributed.

In addition to the changes described above, technology has brought about another structural change, represented by the virtualization of the real environment and the increasingly interactive audio-visual universe permeated by representations and ways of communication that build an electronic hypertext (Castells, 2003, p. 1). The proposed ideal is that of the *enhanced man*, a type of humanity constituted by a virtual *elsewhere* opposite to the real one.

In other words, the whirlwind triggered by technology, which liquefies the cultural foundations underlying the *world of life*, progressively transforms it into the appearance of a *real world* even though it is the actual environment of life. This transition is justified by the need to respond to the needs of man, who must be *enhanced* in his abilities, a character that encloses him in the drift of self-referentiality.

From the scientific-technological perspective, the concept of development changes its meaning and purpose, moving from the engine of growth for man—in his natural endowment—to the engine of growth for technology, to which the human being is bent and turned into a means and not an end. The same body is understood as just something to be used (Garaudy, 1999).

In this trend, there is no convergence of positive interpretations. Garaudy, in particular, proposes a bold experiment to block and reverse the trend: to take on the role of *provocateurs* by intervening in the awareness of (technological) man about the role of science. A paradigm shift is needed that does not view science as an absolute aim and that introduces anthropological purposes within technological processes (e.g., computer-mediated learning).

Martha Nussbaum has also spoken on the subject, and according to her, the current trend will continue: the States of the world will produce generations of docile, useful, and technically qualified machines instead of full-fledged men who can think for themselves, question customs, and understand the sufferings and successes of others (Nussbaum, 2013). At the same time, the political scientist emphasizes that science is permeated by elements of a humanistic spirit: the search for critical thinking, the challenge of imagination, empathy for the most diverse human experiences, and the understanding of the complexity of the world in which we live. Through these remarks, she emphasizes the importance of awareness of the ontological consistency of man and his unity, of his not existing in front of the mere exercise of a self-referential production vs. the distorted and false representation of himself, of others, and of the world (Fornari, 2004).

The outlined cultural climate has developed the fertile humus on which the proposed humanisms have been developed as alternatives to the “classic” one. This is the case of *transhumanism* and *post-humanism*, on which we will dwell in the following pages. With them, there starts again the elaboration of theoretical proposals that point to the heart of postmodernity, a field considered a locus in which the crisis of classical rationality is consumed, leaving no room for anything but contingency, irremovable *differences*, and reductionism.

The scholars of such philosophical configurations try to imagine a model of rationality that does not evade complexity; on the contrary, it engages all its problematicity, arriving at either the virtual, *cyborg*, or enhanced man. A creature pervaded by an intrinsic need to overcome every form of dichotomous thinking, rationality structured around tight dualisms, in favor of a new point of view that enables accounting for an infinitely complex and excess reality, marked by its very presence. The *homo-cyborg*, created by technologies used as extensions of the human body and mind, is based on the hybridization of organic and cybernetic parts, which no longer makes it possible to recognize an intact, *ab origine*, inherent naturalness of the human being. The products of men become an integral part of them, and not just a creation of theirs; they are human beings as well, like their creator.

### 3.1. Transhumanism

Transhumanism is a cultural and philosophical movement that originated in the United States in 1980, following the technological revolution and the spread of information technologies, cybernetics, and nanotechnologies. It brings together scientists from various fields, including philosophers, sociologists, and neuroscientists. The assumptions on which it is based include great confidence in the possibilities provided by science, the consideration of human nature in terms of pure matter, and the reduction of the human mind to mere neuronal connections.

Therefore, transhumanism assumes an ideal of man crushed against his material component, understood as a complex material device characterized by mechanistic operation. The subject is framed in the context of a phase of transition toward the acquisition, activation, or enhancement of physical, intellectual, cerebral, and psychological abilities. From this point of view, which complies with materiality, there is no place to characterize the human being or for the possibility of the existence of something immaterial. Human nature is understood in terms of finalization and rationality where personal reality is traced. Rationalization also presides over the choice of ends, and the decision is made based on criteria of pragmatic utility, with anthropological assumptions assumed as indisputable and universally shared.

Julian Huxley was the first to use the term transhumanism in 1927, referring to technology and its contribution to overcoming human limitations. Nick Bostrom^14^, one of the major theorists of transhumanism, has outlined the characteristic features and objectives pursued by the movement. This new cultural and scientific paradigm projects a futuristic perspective of humanity, embracing the perspectives of modification and improvement of human nature and seeking to structure a body in harmony with desires. The objectives underlying this conception include the expansion of life, the cancellation of the danger of physical and mental deterioration, the implementation of the physical and cognitive capabilities of the human species, and the total emotional control. Therefore, transhumanists are committed to strengthening the limit to allow human beings to pursue their perfection through the enhancement of all their faculties.

The program of transhumanism involves the redesign of humanity through the application of new technologies aimed at eliminating unwanted and unnecessary aspects of the human condition, such as suffering, disease, aging, and even mortality. Additionally, it aims to overcome the limitations of intelligence by creating a psychological profile that is context-dependent and independent of individual will, such as personality pills.^15^

The idea of eliminating biological limitations is an ambitious goal that transhumanists believe is achievable through the integration of man and machine via *mind uploading*^16^, also known as *mind-loading*. This technique involves transferring the entire contents of the human mind onto a digital infrastructure that imitates the brain. For transhumanists, *mind uploading* represents an important possibility for life enhancement, allowing for the achievement of morphological freedom and the overcoming of biological constraints.

Thus, the fundamental idea behind this movement is to technologically improve the human body through the use of prostheses and advances in genetics, biomedical engineering, and nanotechnology. Transhumanist theorists believe that the interconnection of nanotechnologies with biotechnology and information technologies with cognitive sciences will enable the dismantling of the *old* human and bring into existence an individual capable of solving all the problems of the world (Colombo, 2018).

In this scenario, subjectivity resembles the Christian idea of the end of time, after which a new humanity emerges (Bostrom, 2003; Kurzweil, 2013; More, 2013). However, this new humanity may lead to a world in which everything is produced and manipulable, ultimately leading to the end of the world as we know it.^17^

Currently, the process of change pursued by transhumanism appears to be a new utopia or even a modern religious style. It combines techno-optimistic statements from the scientific field, technological research, and the collective imagination of cyber-culture, all converging in a sort of mystical technological development. Only when the *transhumanist revolution*, which supporters see as the new history of man, is no longer a collection of ingenious reflections and good intentions, can it become a unitary system of thought capable of guiding practices and exercising its transforming power over the world.

### 3.2. Post-humanism

Another approach, a refinement of the transhumanism project, is represented by post-humanism. According to its supporters, post-humanism marks a new stage of humanity characterized by an increasingly intense hybridization between man and technology and the end of the opposition between humanism and anti-humanism. This marks a different discursive context compared to previous approaches, as it looks more proactively at new alternatives to anthropocentrism (Braidotti, 2014, p. 44) ^18^.

Post-humanism involves and challenges many of the concepts provided by our tradition. It proposes the renewal of some philosophical assumptions related to the human being, similar to transhumanism, and enhances technological and scientific innovations (Farci, 2011, p. 117–118) mentioned in the previous pages. The technologies that prepare for the advent of the post-human—from genomics to robotics and from informatics to nanotechnologies—are tinged with a strong emotional coloring resulting primarily from the possibility that humans can take control of their evolution, a prospect that creates enthusiasm in some people and great concern in others. Post-humanists consider new technologies as the natural continuation of the cultural process of resolving the relationship between humans and technology. These technologies will allow for the rethinking of the very nature of the human being.

Although a variety of cultural trends converge in this movement, criticism of classical humanism and the purpose of its transformation are pervasive.

Once again, Nietzsche is taken as a nodal reference, particularly his notes on the need to overcome the human being, as expressed in his work *Thus Spoke Zarathustra* (Nietzsche, 2006). From the Nietzschean perspective, humans are old and obsolete, requiring us to go beyond the human creature; humans are beings in transition, not yet a final destination, and humanity is akin to a tightrope between the animal and the superman (the survivor), stretched over an abyss.

Based on these considerations, post-humanists assume as a premise of their approach the crisis of the concept of the human being, which motivates their pursuit of a post-anthropocentric panorama governed by the principles of interaction with other human and non-human agents on a planetary scale, and the need for a human being who can think critically and creatively. They adopt a perspective of life situated “beyond the individual, beyond the species, beyond death”—referring to the title of Braidotti's aforementioned book, one of the greatest representatives of the post-human approach in Italy—and an orientation toward a matter, which is itself vital, intelligent, and capable of self-organization, not dialectically opposed to culture or technological mediation, but close to them, which will allow the overcoming of human decadence (Braidotti, 2014).

Katherine Hayles, in *How We Became Post-human*, clarifies the process behind the new transformative dynamics of the human being. The scholar argues that the rise of information technology has initiated the dematerialization of bodies and encouraged the progressive abandonment and transcendence of the constraints of materiality (Hayles, 1999).

On the other hand, for Braidotti, it is a fact that we are all post-humans, and she supports this statement with the evidence of the modifications already visible in a body transformed into a connected object, a nano bio-info-neuro machine, an announcement of the end of the human being in traditional forms (Braidotti, 2014). The Italian scholar assumes as the basis of her theory on the post-human, a hybrid construction of humans made possible by advances in science and technology (informatics, biology, and bioinformatics), which allows for the overcoming of the traditional conception of the human body as pure materiality. She believes that a new species is being created with the help of more efficient artificial supports, which would validate what has already been suggested by Moravec (1999), Chislenko (2011), and More (2013): the advent of robot-men.

This vision of humanity is supported by the emergence of new *skills*, awareness, knowledge, and techniques that have brought about an urgency to push beyond the limits that define the human being. This paradoxical idea is justified by the belief in human *perfection* achievable through the strengthening of human faculties.

One of the leading advocates of post-humanism internationally is Donna Haraway, who argues in her book *A Cyborg Manifesto* that the theory of the cyborg was inspired by social arguments, particularly the issue of feminism (Haraway, 2011). The American philosopher considers the tendency of human beings to rebuild themselves through technology to differentiate themselves from other biological forms on the planet as a natural tendency. Humans have always sought to modify the human body, a project that continues today through the use of technological prostheses and genetic engineering through the modification of the organism's genes. According to Haraway, the desire to improve nature's design is at the root of human culture, and in the postmodern era, we all live in a mythical time where we are all cyborgs—hybrids mechanized and manufactured by machines and organisms (Haraway, 1991).

Therefore, the cyborg opens up a new ontology as a condensed form produced both by the imagination and material reality, two united centers that create every possibility of historical transformation. Although the relationship between organism and machine has been rejected in the tradition of Western science, the cyber-body, empowered by its performance, has allowed for the overcoming of the post-industrial production system. This is a body capable of reinventing or reorganizing technological development, on which scientific debate and cultural imagination converge (Macrì, 1996, p. 10).

Donna Haraway continues her narrative about the change taking place by arguing that there is no human identity without a social context and that we are all subject to change, which becomes clear from observing the cyborg, a creature made through technical power that embodies a new form of human identity. Braidotti also contributed to the new conception of identity, defending the idea of a nature-culture continuum, and referring to a relational subject determined by the constitutive multiplicity of a nomadic subjectivity, thereby surpassing the unitary subject (a reference to classical humanism).

According to post-humanism, the subject is a *mobile assemblage*, a transversal entity, fully *immersed in* and *immanent to* a network of non-human relationships, from animal relations to plants and viruses, including the technological apparatus. It is more than just a subject; it refers to an *artificial otherness*, which is also the subject of literature (such as Asimov) and cinema (such as Blade Runner by Ridley Scott). These areas have raised issues related to the esthetics and identity of the replicating robot, as well as questions related to ethics and the integration of moral laws into virtual beings.

## 4. Bankruptcy of humanism: toward a secular Humanism and “enhanced man”?

Based on the reflections presented, it is evident that there is an attempt to deconstruct humanism. Transhumanism and post-humanism, cultural movements united by a shared vision that eliminates any nostalgia for the human being or regret for the individual, subject humanism to a frontal attack and reduce the individual to functional rationality. This understanding of human nature contains a questionable element: the inability to comprehend the sense of ontological dignity that is proper to every human being.

The removal of the ontological foundation, the essence that differentiates humans from other living beings, introduces a materialistic reductionism with dangerous consequences. By pursuing the transformation of the person into an object, like other things, it introduces a quantitative ontological egalitarianism that distinguishes humans from animals, objects, and even super-intelligent machines due to greater quantitative complexity. In this way, the peculiar characteristics of human essences, such as dignity, are lost and become expressions of subjectively defined meanings like the quality of life and capacity for autonomy.

In other words, transhumanism and post-humanism propose a new conceptualization of the human being that challenges the (Western) humanistic philosophical tradition and seeks to abandon the previous anthropological vision. To achieve this goal, they deconstruct the previous oppositional duality between nature and culture and between human and non-human, and view humans not merely as users of technological devices, but as entities that incorporate technology to amplify their capabilities, for example, overcoming space–time obstacles.

In the end, these approaches identify themselves with the attack launched by the scientific-technological domain against the human being, based on the advances of science and computer technology, biology, and bioinformatics. They propose and seek to realize overcoming the concept of the body as integral to man, assuming corporeality as an entity constituted by artificial functional supports to enhance efficiency and achieve immortality, two characteristics peculiar to the new human species.

From the aforementioned reflections, it becomes clear that there is an attempt to deconstruct Humanism. This philosophy is facing a direct attack from transhumanism and post-humanism, cultural movements that share a vision of removing any nostalgia or regret for the human being as an individual, reducing him to functional rationality, and thus misunderstanding the individual. This way of understanding the perceived nature of man contains a questionable element: the inability to comprehend the ontological dignity that is proper to every human being.

The removal of the ontological foundation, the essence that differentiates man from other living beings, introduces a materialistic reductionism with dangerous consequences. Pursuing the transformation of the person into an object like other things introduces a quantitative ontological egalitarianism whereby man is distinguished from animals, objects, and even super-intelligent machines due to greater quantitative complexity. In this way, he loses the peculiar characteristics of his essence as a person, such as a dignity, which becomes the expression of subjectively defined meanings (quality of life, capacity for autonomy, etc.).

In other words, transhumanism and post-humanism propose a conceptualization of the human being that challenges the (Western) humanistic philosophical tradition and aims to abandon the previous anthropological vision. They deconstruct the previous oppositional duality between nature and culture and between human and non-human, and assume the ideal of man not as a simple user of technological devices but as an entity that incorporates them to amplify his own capabilities (e.g., overcoming space–time obstacles).

Ultimately, these approaches identify themselves with the attack launched by the scientific-technological domain against the human being based on the advances of science and computer technology, biology and bioinformatics, and their proposal and realization to overcome the concept of the body as integral to man. They propose the overcoming of the approach to a body made of flesh and bones, assuming the corporeality as an entity constituted by artificial functional supports to implement his efficiency and immortality, two characteristics peculiar to the new human species.

An ideal that finds enthusiastic supporters of a future inhabited by men who transform themselves into robots, distinguished, however, from the machine by a sort of soul that can transit through different bearers, as mentioned by Hans Moravec, Max More, Alexander Chislenko, etc.

In the new cultural perspective, the destiny of man is to become a cyborg, an icon that moves from the imaginary to the real. Transhumanism and post-humanism propose scientific advances that were once believed to be materials for literary and science fiction models (e.g., Philip K. Dick and William Gibson) conveying the ideal of hybridization. This is a perspective that has its roots in ancient myths, starting with Frankenstein and ending with the current cyborg. All are united by the combination of organic and cybernetic parts and the impossibility of recognizing an inherent naturalness, ab origine, of the human being.

In this cultural climate, the relationship between the human being and technical power is fundamental for the transformation into reality of the Superman, the Übermensch (Haraway, 2011), a myth present throughout history.

The two cultural movements come up with a simplified and fragmented version of the human being by claiming that they want to promote the liberation of man, more precisely, to set man free from limitations, cultural identities, ideologies, etc. They pursue their goal by neglecting the fact that the human being is inseparably connected with questions of meaning and does not represent just a stage for the infinite and equivalent possibilities of manipulation of himself.^19^

The perspective of transhumanism and post-humanism implies a future in which the human being, by building himself up, will become whatever he desires, regardless of comparisons with anthropology, ethics, or what scientific progress can reasonably determine. These two movements present a drift of abstraction proper to contemporaneity, contradicted by the fact that beyond his techno-economic efficiency, man remains what he has always been: a lack of being and desire for others.

Numerous scholars have expressed their disagreement in this regard. Fukuyama, for example, defined transhumanism as “one of the most dangerous ideas in the world” because it alters human nature and the concept of total equality among all human beings, which is the foundation of every democratic society (Fukuyama, 2003). Habermas has criticized the theory and assumptions of transhumanism and post-humanism enhancement because they would eliminate the possibility of moral autonomy of the human being, who would then become submissive to social, political, or economic interests.

This is not intended to ignore the fact that the refinement of technology and its possibility of use has helped achieve previously unimaginable goals in the medical and rehabilitation field, as well as in engineering, physics, architecture, and entertainment in general, producing new professional figures and greater satisfaction of end users, from patients to consumers. This has helped give a new (and necessary) impetus to the innovation industry. These results show that correct and conscious management of these resources can help optimize the status quo of human beings without incurring excessive distortion and evolutionary risks.

The advanced technological development context we are experiencing, characterized by rapid and pervasive digitalization, questions our traditional conceptions of what it means to be human. The schools of thought of transhumanism and post-humanism offer extreme and sometimes distorted visions of the human being, which might be seen as a threat to our very humanity. Nevertheless, a return to a past Humanism, a return to an idealized and romantic view of the human being, is impractical and not adhering to the current reality.

In his book *Funzionare o esistere* (Benasayag, 2019a), the philosopher and psychoanalyst Miguel Benasayag addresses the issue by proposing an alternative perspective. Instead of opposing the current of the technological era and trying to return to an inaccessible past, he suggests adopting a more pragmatic and dynamic attitude, characterized by an irreversible negotiation with machines. The central point of this vision is the recognition that the hybridization between humans and technology has already occurred and is a fact of our current society. Instead of resisting this reality, we should seek ways to coexist and interact with technology in a way that preserves and enriches our humanity.

Benasayag's perspective implies recognition and appreciation of every individual's singularity. In this view, each person is seen as unique, with their qualities and fragility, and the relationship between individuals is considered a fundamental element of human society. This conception contrasts with the vision of transhumanism and post-humanism, where human individuality may be seen as something to overcome or transcend through the use of technology.

Therefore, it is not about opposing a romanticized humanity to technological progress but seeking a dynamic balance where technology and humanity can coexist and thrive. This means finding ways to use technology that respect and value our humanity, rather than trying to transcend or eliminate it. In the age of digitalization and automation, this is a challenge that requires profound critical thinking and ethical reflection.

The question, therefore, shifts from opposition to constructive coexistence between the human and the machine. This coexistence should not be seen as a threat but as an opportunity to enrich our way of being human, valuing our singularities.

It is essential, however, to distinguish between adopting technological tools that improve the quality of life and uncritically accepting a technocentric vision of existence. The use of technology can and must serve humanity, but must never lose sight of the importance of individuality, autonomy, and human dignity.

This vision emphasizes the need for continuous and critical dialogue with technology. It is not about rejecting technology, but understanding how we can use it in a way that respects our humanity. This involves ongoing investigation into the ethical implications of technology use and its interaction with our fundamental values.

Benasayag's proposal further requires recognition of relationality as an integral part of our humanity. Relationships between individuals are not just an accessory aspect of our existence but constitute an essential element. In an increasingly connected and interdependent society, the ability to build and maintain positive and meaningful relationships becomes increasingly important.

Finally, the vision of a New Humanism, as suggested by Benasayag, requires an active commitment to shaping the direction of our future. We cannot remain passive in the face of advancing technology, but must actively seek to influence its development in a way that respects and values our humanity. This requires broad participation from all social actors and from the scientific and technological community to the political world and education, for each individual.

In conclusion, the idea of a New Humanism, as proposed by Benasayag, is neither a rejection of technology nor a return to an idealized past, but rather an invitation to critical reflection and an active commitment to creating a future where technology and humanity can coexist constructively and enrichingly.

## Author's note

The verb “to slouch” is employed by Nina Coltart, referring to the poem “The Second Coming” by William Butler Yeats, written in 1919 and translated into Italian as “arrancare.” The well-known line that incorporates this word reads: “And what rough beast, its hour come round at last/Slouches toward Bethlehem to be born?”

## Author contributions

All authors listed have made a substantial, direct, and intellectual contribution to the work and approved it for publication.

## Footnotes

^1^The version that takes on the connotations of minimalist narcissism.

^2^This paradox urges the Italian Church today to be at the forefront of the search for New Humanism.

^3^The anthropological premises underlie the development and organization model of social life.

^4^Whenever human beings discriminate against one another, it occurs due to a lack of universality, rather than an excess of it. The defense of human integrity cannot be detached from the sustainability of the environment, economy, and society. This is because the values that need safeguarding on a personal level (such as life, family, and education) are vital for the protection of social values (like justice, solidarity, and work) as well.

^5^Why should human beings exist in the world? This is the question to which the new ethics is compelled to provide an answer, and one that renders traditional ethics insufficient in the eyes of Jonas. The technological advancements urge us to reexamine the age-old inquiries concerning the relationship between existence and purpose. Consequently, the new ethics, in contrast to modern subjectivism, must not solely focus on humanity but contemplate the long-term consequences of our actions. According to Jonas, the new ethics must reject both ruthless anthropocentrism and the structural myopia inherent in traditional ethics rooted in Hellenistic and Jewish-Christian traditions. This implies that it is no longer adequate for us to find solace within our conscience or adhere to formal rules alone (such as those found in religious teachings), but we must possess the ability to foresee the potential impact our actions might have on the fate of humanity and the planet.

^6^Regarding the distinction between the individual and person, it is necessary to clarify when discussing humanity.

^7^Ulysses, apart from his cunning intellect (even if utilized for deceptive purposes), serves as an example of an insatiable thirst for knowledge and an irrepressible curiosity that drives him to venture into the realm of the unknown.

^8^The question still remains unresolved as to whether man, on his own, can adequately meet the inherent need for knowledge that defines human beings and truly exert control over their own destiny and the course of events. The answer lies within medieval culture, which encompasses a fundamental connection between the greatness of man and the grace of God.

^9^In this regard, it would be valuable to revisit the lesson of the Gospel that highlights a transformative power, exemplified by the case of Rome. With Constantine's conversion to Christianity and his subsequent reign, the world underwent significant changes over a span of 150 years, achieved without the use of military force.

^10^In contemporary Italy, Humanism itself, originally influenced by Christianity, has taken shape as a pluralistic phenomenon. Various individuals, with diverse backgrounds, states of life, cultural foundations, and spiritual sensibilities, have contributed to its development. This ranges from great saints to numerous individuals engaged in charitable endeavors, educational work, as well as cultural, social, and political spheres of involvement.

^11^Conception that contrasts with the tension, inherent in all cultures, the idea of the man able to bypass the particularistic and one-dimensional visions (e.g., individualism and narcissism).

^12^Zygmunt Bauman provided us with an enlightening intuition: the concept of *backstop*: “We have reversed the course, and we are sailing backwards. The future is in the stocks and the past has been moved between credits, it has been either rightly or wrongly reassessed, where hopes are not yet discredited. These are the years of retrotopia” (Bauman, 2018). Therefore, if utopia is the visionary impulse toward a dreamed future as the resolution of the contradictions of the present, the desire-project of a better society, the retrotopia is, in the age of fear, the nostalgia of the past, the refuge in the established securities of yesterday; and it is undeniable that our present is sick with retrotopia.

^13^Technology is mainly used for an exchange of interest, for mutual enjoyment, to establish relationships, while in schools, it is used to replace chalk with tablet. On the other hand, Recalcati proposes to place the teacher's word at the center of the lesson time, an *event* that breaks out when the scene is occupied by knowledge screwed on free presumptions of possession.

^14^Nick Bostrom is a professor at Oxford University, where he serves as the founding director of the Future of Humanity Institute. He is also the president of the World Transhumanist Association (WTA). All of his articles and publications can be found at: http://www.nickbostrom.com.

^15^The goal of personality pills is to modify personality to overcome limitations such as shyness or enhance creative and emotional capacity.

^16^Mind transfer, also known as mind uploading, is the hypothetical process of transferring or duplicating a conscious mind from a biological brain to a non-biological substrate. This process entails scanning and creating a detailed map of the biological brain, and then replicating its state onto a computer system or other computational device. The computer would run a simulation model that closely resembles the original mind, behaving in a manner indistinguishable from the original brain, at least for all practical purposes. This simulated mind would be considered a part of the simulated world's reality and could be supported by a three-dimensional anatomical model that simulates a body. Alternatively, the simulated mind could inhabit a computer or be connected to a humanoid robot or biological body, effectively replacing the original brain. Mind uploading serves as a central theme in various science-fiction works, including novels and films like *Transcendence*.

^17^The superficial treatment of a delicate issue such as gender is indicative of the arrogance that can affect technologically driven individuals.

^18^According to Braidotti's “post-human criticism,” the discussion of norms, values, community ties, social affiliations, and political governance inherently relies on the concept of a subject (Braidotti, 2014, 50). This presumed subject ought to possess the courage to embrace and transform their existing state, to gain a comprehensive understanding of their present potential, whether consciously or subconsciously.

^19^There is no longer just man replacing God, but even the denial of God's place. Such solution is unrealistic and unsustainable, and generates contradictory adjustments, such as the escape into a lush imagination that avoids confrontation with reality. Or the “fundamentalist (or populist) reaction,” a “hysterical” response to fragmentation through the attachment to rigid and, therefore, rancorous and violent simplifications (Magatti, 2015).
